# Diabetes self-care activities and health-related quality of life of patients with type 2 diabetes in Ho, Ghana: a cross-sectional study

**DOI:** 10.1186/s12902-025-02067-z

**Published:** 2025-11-11

**Authors:** Stanley Kofi Alor, Irene A. Kretchy, Franklin N. Glozah, Philip Baba Adongo

**Affiliations:** 1 Department of Social and Behavioural Sciences, School of Public Health, University of Ghana, Legon, Accra Ghana; 2 Department of Pharmacy Practice and Clinical Pharmacy, School of Pharmacy, University of Ghana, Legon, Accra Ghana; 3 Ghana Armed Forces College of Nursing and Midwifery, 37 Military Hospital, Neghelli Barracks, Accra, Ghana; 4 Family Health Medical School, Family Health University, Teshie, Accra Ghana

**Keywords:** Type 2 diabetes, Health-related quality of life, Diabetes self-care activities, Glycemic control, Ghana

## Abstract

**Background:**

Adherence to diabetes self-care activities (DSCA) has been recognised as a major factor to reduce complications and improve the health-related quality of life (HRQOL) of patients with diabetes. However, despite the increasing cases of diabetes and its complications in Ghana, there are few or no studies in Ghana that have explored how sociodemographic and clinical factors influence diabetes self-care activities and HRQOL among patients with type 2 diabetes. This study aimed to assess sociodemographic and clinical variables influencing the performance of DSCA and its influence on HRQOL of patients with type 2 diabetes in Ghana.

**Methods:**

This is a hospital-based cross-sectional study conducted among adults (N=310) with type 2 diabetes mellitus who were accessing healthcare at two public hospitals in Ghana. The performance of DSCA and HRQOL was examined using the summary of the Diabetes Selfcare Activities (DSCA) Questionnaire and the EQ-5D Questionnaire for HRQOL. The differences and associations between the variables were analysed using the chi-square test, multiple ANOVA, t-test, and Tukey’s HSD for post-hoc tests. -based.

**Results:**

The participants were more likely to adhere to medication and a healthy diet and less likely to adhere to self-monitoring of blood glucose levels and foot care. Most patients adhere to medication and diet, but less to foot care and blood glucose monitoring. Men and patients with diabetes education reported higher adherence to self-care activities.

**Conclusion:**

Diabetes education and regular monitoring of diabetes self-care activities are essential to improve HRQOL in patients with type 2 diabetes in Ghana.

## Introduction

Type 2 diabetes mellitus (T2DM) is a serious chronic metabolic disease that occurs in adolescents and adults when the body is unable to use insulin effectively. The prevalence of T2DM is increasing globally, particularly in Africa [1]. Type 2 diabetes can cause damage to many of the body’s organs, leading to disabling and life-threatening health complications such as cardiovascular diseases, nerve damage, kidney damage, lower-limb amputation, and eye disease mainly affecting the retina, resulting in loss of vision and even blindness, cognitive decline, liver disease, cancer, and frailty [1, 2]. Type 2 diabetes mellitus is the most common type of diabetes, accounting for over 90% of all diabetes cases and the seventh leading cause of disease burden globally [2]. Diabetes has reached pandemic levels, with a prevalence rate of 10.5% of adults between the ages of 20 and 79 years [1]. A 2025 study by the International Diabetes Federation (IDF) to investigate the cases, deaths, prevalence, causes, prevention and cost of care for diabetes globally has found that 3.4 million adults have died from diabetes-related complications in 2024, corresponding to 9.3% of global deaths from all causes [2]. Additionally, diabetes cases have increased from 200 million in the 1990 s to 589 million in 2024, with 10.5 prevalence rate. With the causes, the study has found that increasing urbanisation, physical inactivity, and unhealthy diet [2] were the risk factors. The global expenditure on diabetes has been considerable, growing from USD 232 billion in 2007 to more than a trillion USD in 2024 for adults, representing a 338% increase in the last 17 years and representing 12% of global health expenditure in 2024 [2]. The study also found that the prevalence of diabetes was 4.2%, with 24.6 million people living with the disease in Africa. The estimates have shown that Africa would bear the highest increase in diabetes cases of 142%, by 2050, compared to 10% in Europe [2].

Ghana is one of the countries with a high number of diabetes cases (about 2.5 million cases) in the African region of the International Diabetes Federation, with a prevalence rate of 6.4%, higher than the African prevalence rate of 4.2%, with increasing emergency medical admissions in Ho among patients with type 2 diabetes possibly due to inadequate daily diabetes self-care activities [2, 3]. Additionally, there are many people having impaired glucose, an indication that they are at high risk of developing T2DM in their lifetime [4]. A 2025 study in Ghana has found that between USD 373 and USD 1,207 is spent on diabetes care [5]. Diabetes self-care is important to maintain optimal glycaemic control and prevention of complications, which can have adverse effects on health-related quality of life (HRQOL) and have fatal consequences [6]. A significant association has been reported among patients with diabetes in performing DSCA and HRQOL in Asia [6, 7] and Africa [8]. Diabetes self-care activities (DSCA) require patients with diabetes to adhere to medication and a healthy diet, frequent monitoring of blood glucose levels and foot care and participation in regular physical activity https://www.frontiersin.org/journals/clinical-diabetes-and-healthcare/articles/10.3389/fcdhc.2021.823046/full. [9]. However, over 50% of patients with diabetes did not take their diabetes medication in 2022 [2]. Inadequate attention to diabetes, especially in low- and middle-income countries, including Ghana, has resulted in preventable deaths and disabilities [10]. These preventable deaths and disabilities can be avoided or delayed with DSCA [11].

The psychological and emotional wellbeing of patients with T2DM and their families is adversely affected [6]. Health-related quality of life assesses the individual’s perception of health and is a useful measure of Disability Adjusted Life Years (DALYs). The HRQOL is a multidimensional concept, comprising physical, emotional and social components [9]. The main outcome measure in T2DM may not be glycaemic control but perceived HRQOL, which is paramount in diabetes health outcomes as reported by many studies [11–14]. Patients with T2DM may have varying HRQOL based on their daily activities, sociodemographic and clinical variables [6]. Furthermore, diabetes self-care is associated with metabolic control; however, there is no data on which of the DSCA have influence on glycaemic control and HRQOL [14, 15].

However, only a few studies in Ghana have explored how sociodemographic and clinical factors influence DSCA and HRQOL among patients with type 2 diabetes. Therefore, understanding the sociodemographic and clinical factors influencing DSCA and HRQOL would help healthcare providers design appropriate interventions to improve the wellbeing of patients with T2DM. The aim of this study was to assess DSCA and HRQOL of individuals with T2DM accessing healthcare at Ho Teaching and Municipal Hospitals in Ghana.

This study was underpinned by the health belief model (HBM) conceptualised in the 1950 s to help understand preventative health behaviour [16]. It has been applied in chronic disease prevention to health education and promotion. The model helps to understand the failure of patients with diabetes to perform DSCA to help improve their HRQOL. The model consists of six dimensions that influence behaviour: perceived susceptibility, perceived severity, perceived benefits, perceived barriers, self-efficacy, and cues to action.

## Methods

### Study design, setting and sample size

**Fig. 1:**
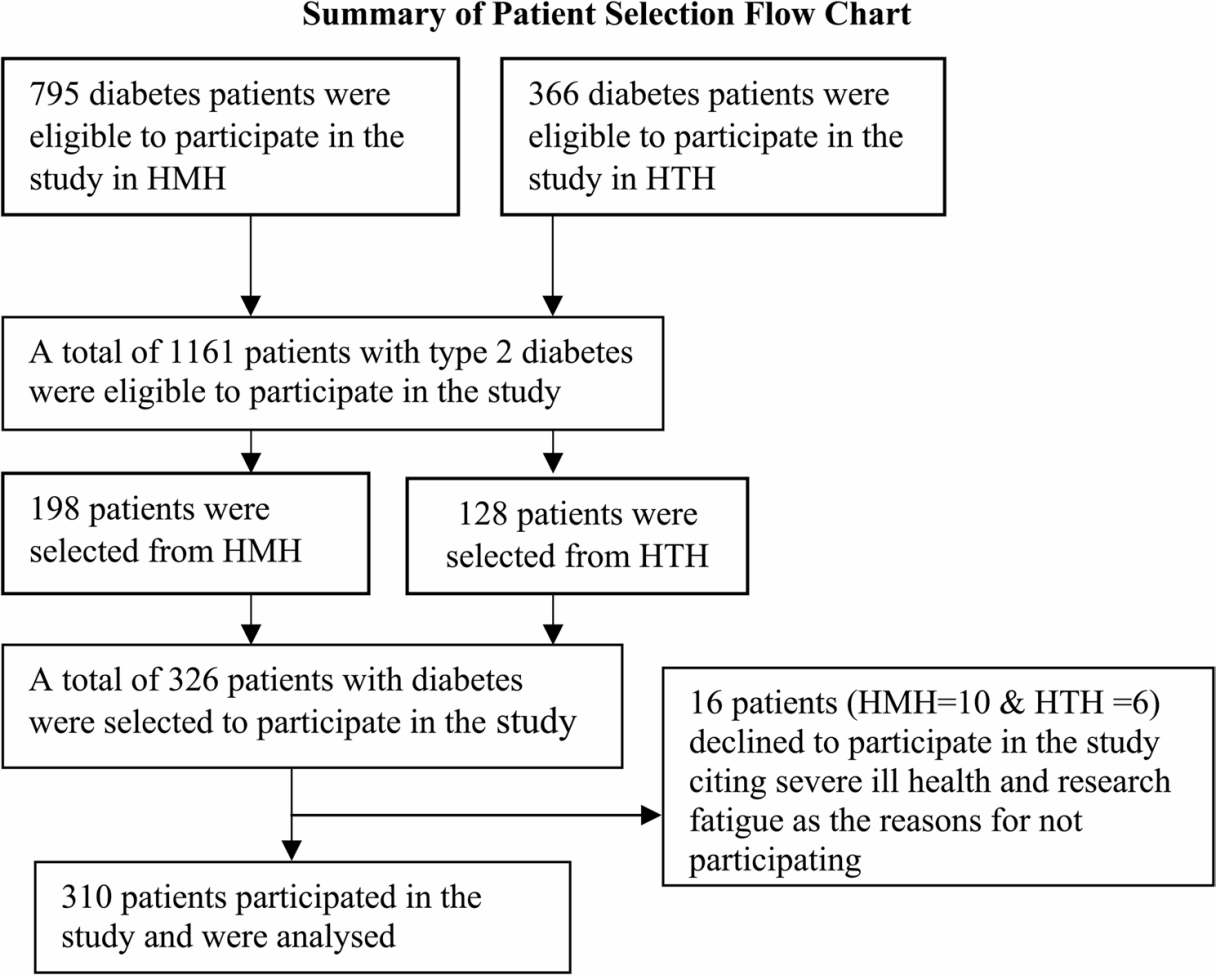
Summary of flowchart record selection, 2022

This is a hospital-based cross-sectional descriptive study conducted in two public hospitals in Ghana; Ho Teaching Hospital (HTH) and Ho Municipal Hospital (HMH) are located in the Volta regional capital, Ho, about 156 km from the capital city of Ghana, Accra. These hospitals were selected for the study due to increasing medical emergency admissions of patients with T2DM. The people are predominantly peasant farmers and traders. The two hospitals serve as primary and referral centres for an array of diseases, including diabetes, from the region and beyond. The two hospitals serve more than 400,000 patients per year. A review of patients’ charts at both hospitals indicated that a total of 1,161 patients (HMH-795 and HTH-366) with T2DM were accessing healthcare at two hospitals at the time of data collection, as represented in Fig. 1. The number of patients accessing healthcare at HMH was more than that of HTH. This is because HMH operates the diabetes clinic from Monday to Friday for patients to access diabetes care while the HTH operates the diabetes clinic on Thursdays only, and therefore more patients tend to HMH since both facilities provide the same diabetes care and education services. Using the Yamane formula (1967) [17], N/1 + Ne^2^ with a 95% confidence interval, a 5% margin of error and a 10% non-response rate, a sample size of 326 was determined. Using systematic random sampling, the total population was divided by the total sample size, i.e., 1161/326 = 3.5 and rounded up to 4. The charts of patients were arranged in the order in which they came to the clinic, and the starting point was determined randomly, and from that determined number, every 4th patient was recruited to participate in the study until the last participant was recruited. However, 310 patients with T2DM participated in the study, and their data were analysed, while 16 patients declined to participate in the study.

### Inclusion and exclusion criteria

Patients who were (i) clinically diagnosed with T2DM for at least 12 calendar months; (2) living in the Ho municipality; (3) aged 18 years and above; (4) willing to sign an informed consent form and (5) accessing healthcare at HMH or HTH were recruited to participate in the study. Patients who were seriously ill, newly diagnosed patients with T2DM and pregnant women with T2DM were excluded from the study.

## Measures

Following permission from the HTH and HMH, a standard questionnaire was administered to patients with T2DM to collect data concurrently from both hospitals from April to September 2022. The questionnaire covered three sections: (i) demographic characteristics; (ii) diabetes self-care activities; (iii) health status of patients. The study participants were asked about their age, sex, marital status, religious affiliation, place of residence, educational attainment, monthly income, education on diabetes, and review of patients’ charts for the heights and masses of the patients for calculating Body Mass Index (BMI), glycaemic control, and duration of diabetes. The questionnaire was administered to the participants ’digitally using the CSEntry app for data collection.

The Diabetes Self‑care Activities (DSCA) [18] consist of a 15-item self-reported questionnaire with five domains. The DSCA has been used to study DSCA in Ghana [19], China [6], Pakistan [7], and Ethiopia [20]. The five dimensions of the DSCA are: (1) diet measured by four items; (2) exercise measured by two items; (3) blood glucose monitoring measured by two items; (4) foot care measured by five items; and (5) adherence to medications measured by two items in the past 7 days prior to data collection. These subscales were measured on a seven-point Likert scale response format ranging between 0 and 7. The mean scores on the items were computed for the five scales to obtain the subscale scores for each domain. The sum of the subscales creates a composite score for total diabetes self-care. The reliability of the questionnaire is within an acceptable range, with Cronbach’s alpha ranging from 0.72 to 0.96 for all five subscales. The overall internal consistency was 0.96 for this study instrument.

The EuroQol EQ-5D Questionnaire [21] was used to measure the health status of patients [21, 22]. The EQ-5D questionnaire has been applied to a wide range of health conditions and treatments, as it provides a simple descriptive profile and a single index value for health status that can be used in the clinical and economic evaluation of health care as well as in population health surveys [21, 23–25]. The HRQOL of patients with T2DM in this study was assessed using the EQ-5D-3 L [21]. Participants classified their HRQOL status at three levels: not affected at all (none), moderately affected, and severely affected in five dimensions of HRQOL (mobility, self-care, usual activities, pain or discomfort, and anxiety or depression), resulting in scores that have been converted into a single index value for health status (1 = full health, 0 = health affected) [21, 22, 26]. Additionally, participants completed the EQ-VAS scale to rate how well or bad their health status was [6, 21, 27]. The internal consistency for the EQ-5D-3 L questionnaire in this study was 0.79. ‘Clinical trial number: not applicable.’

### Ethical considerations

Ethical approval was granted by the Ghana Health Service Ethics Review Committee with ethics number GHS-ERC: 003/03/22. All procedures contributing to this study complied with the guideline on human experimentation and with the Helsinki Declaration of 1975, as revised in 2013 [28].

### Statistical analysis

The data were presented as frequency (n), percentages or mean ± SD. The chi-square test was used to examine the differences and associations between the variables, multiple ANOVA to compare the means across education and employment levels, Tukey’s HSD for post-hoc tests to determine the means that are statistically different from one another, and the t-test to compare the means of sex and diabetes education in the DSCA performance [29, 30]. A single-step multiple comparison test was performed to investigate where the difference lies in the mean score. Independent variables included age, sex, marital status, employment type, educational attainment, diabetes duration, monthly income, residence (urban or rural), and diabetes education, and the dependent variables were DSCA and HRQOL. Statistical analyses were performed using STATA version 15.0. The significant variables were presented in the tables. A *p* ≤ 0.05 was considered statistically significant.

## Results

The results in Table 1 show the socio-demographic and clinical characteristics of the study participants. Out of 310 participants, 204 (65.8%) are females. The age of the participants ranged from 18 to 85 years, and the majority of the participants, 164 (53%), were between the ages of 56 and 75 years. Also, 111 (35.8%) of the study participants had junior high school. The majority of the respondents were married, 194 (62.6%), and 132 (42.6%) of the participants were self-employed. Also, 193 (62.3%) of the participants lived in urban areas, and 74 (23.9%) of the participants earned income between GH501 and GH1000 per month. Also, 86 (28.0%) of the participants had good glycaemic control. The mean BMI of the participants was 25.4 (± 4.38) kg/m^2^, and 146 (47.1%) of the study participants were overweight. The results also show that 127 (41.0%) of the participants had been living with diabetes between 1 and 5 years, and a majority of the respondents, 273 (88.1%), have had diabetes education. Additionally, 227 (73.2%) of the patients do not have personal glucometers, 212 (68.4%) have diabetes complications and 217 (70.0%) have diabetes comorbidity.

**Table 1:** Socio-demographic and clinical characteristics of patients with Diabetes in Ho (*n*=310)

Social Demographic Characteristics	Frequency (*n*)	Percentage (%)
Age group (years)		
25–35	13	4.2
36–55	118	38
56–75	164	53
76–85	15	4.8
Sex		
Male	106	34.2
Female	204	65.8
Marital status		
Not married	24	7.7
Married	194	62.6
Divorced	26	8.4
Widow	66	21.3
Religion		
Christian	295	95.2
Moslems	15	4.8
Educational Level		
Primary	82	26.4
Junior High School (JHS)	111	35.8
Senior High School (SHS)/VOC/TECH. Sch.	34	11
Tertiary	83	26.8
Employment status		
Unemployed	123	39.6
Public sector	43	13.9
Private	12	3.9
Self-employed	132	42.6
Residence		
Rural	117	37.7
Urban	193	62.3
Monthly income GH¢		
No income	101	32.6
1–500	48	15.5
501–1000	74	23.9
1001–1500	42	13.5
1501–2000	18	5.8
≥ 2001	27	8.7
Diabetes Education		
Yes	273	88.1
No	37	11.9
Duration of Diabetes (years)		
1–5	127	41.0
6–10	114	36.7
11–15	43	13.8
16–20	17	5.5
≥ 21	9	3.0
Glycemic control (HbA1c)		
Good glycemic Control < 7.0 mmol/L	86	28
Poor glycemic control ≥ 7.0 mmol/L	224	72
Body Mass Index (BMI)		
Underweight < 18.5 Kg/m^2^	5	1.6
Normal 18.5–24.9 Kg/m^2^	67	21.6
Overweight 25.0–29.0.9Kg/m^2^	146	47.1
Obese ≥ 30.0	83	26.8
Morbid obese ≥ 40 Kg/m^2^	9	2.9
Personal Glucometer for monitoring blood glucose		
Yes	83	26.8
No	227	73.2
Diabetes Complication		
Yes	212	68.4
No	98	31.6
Diabetes comorbidity		
Yes	217	70.0
No	93	30.0
*USD 1=GH¢13.53		

The summary of DSCA among patients with T2DM in Ho, Ghana is presented in Table 2. From the analysis, participants were more likely to adhere to medication and healthy diet compare to self-monitoring of blood glucose levels and foot care.

**Table 2:** Summary of DSCA among patients with T2DM in Ho, Ghana = 310

Data set	Values
Diabetes Self-care Activity (DSCA)	Means ± SD
Healthy diet	3.03 ± 1.0
Physical activity	2.24 ± 1.6
Self-monitoring of blood glucose (SMBG)	1.22 ± 1.6
Foot care	1.83 ± 1.6
Medications adherence	3.97 ± 1.4
Total	2.46 ± 0.7

In relation to the summary of HRQOL, presented in Tables 3 and 125 (40.3%) have reported moderate mobility difficulties during the period, 88 (28.4%) were moderately affected in providing self-care, and 140 (45.1%) were moderately affected in performing usual activities. Additionally, 220 (71.0%) participants had experienced moderate pain or discomfort, and 178 (57.4%) also experienced moderate anxiety and depression.

**Table 3:** Summary of HRQOL (EQ-5D) among patients with T2DM in Ho, Ghana = 310

DIMENSIONS OF HRQOL (EQ-5D)	*N* (%)
Mobility	
Not affected	170 (54.8)
Moderately affected	125 (40.4)
Severely affected	15 (4.8)
Self-care	
Not affected	200 (64.5)
Moderately affected	88 (28.4)
Severely affected	22 (7.1)
Usual activities	
Not affected	153 (49.4)
Moderately affected	140 (45.1)
Severely affected	17 (5.5)
Pain/discomfort	
No pain/discomfort	64 (20.6)
Moderate pain/discomfort	220 (71)
Severe pain/discomfort	26 (8.4)
Anxiety/depression	
No anxiety/depression	113 (36.5)
Moderate anxiety/depression	178 (57.4)
Severe anxiety/depression	19 (6.1)
Your health today (EQ-VAS)	70.65 ± 13.5
EQ −5D index	082 ± 0.2
EQ-VAS = visual analogue scale	

However, some participants, 26 (8.4%) and 19 (6.1%), have reported severe pain and discomfort and anxiety and depression during the period. Furthermore, 17 (5.5%), 22 (7.1%) and 15 (4.8%) of the participants reported that they were severely affected in performing usual activities, self-care roles and mobility difficulties during the period, respectively.

From the simple T-test in Table 4, male patients with T2DM scored about 0.2 points higher in the performance of DSCA compared to female patients. This was statistically significant at *p* < 0.03. Similarly, patients who had received education on diabetes scored 0.6 points higher in performing DSCA compared to patients who had not received education on diabetes. This was found to be statistically significant at *p* < 0.001, as presented in the simple T-test in Table 4.

**Table 4:** Simple T-test

Variables	Means	Standard Error	*p*-value
Sex			
Male	2.58	(0.06)	
Female	2.40	(0.05)	
Combined	2.46	(0.04**)	0.03
Education on Diabetes			
Yes	2.5	(0.04)	
No	1.9	(0.09)	
Combined	2.5	(0.04**)	0.001

** for 5%

A multiple ANOVA was performed to compare the effects of the educational attainment of patients on the performance of DSCA and found that there was a statistical difference in the mean score in the performance of self-care activities among the patients’ educational attainment (F (5, 304) = 7.42, *p* < 0.001). Tukey’s HSD test for multiple comparisons found that the mean score for patients with tertiary education was significantly different from patients with primary and JHS-level education.

Similarly, comparing the effects of the type of employment of patients with T2DM on the performance of DSCA, the results showed that there was a statistical difference in the mean score for the performance of DSCA among the respondents (F (5, 304) = 14.22, *p* < 0.001). Tukey’s HSD test for multiple comparisons found that the mean score for patients in public sector employment was significantly different from that of self-employed and unemployed patients. Additionally, patients who were employed in the private sector had higher mean scores for self-care activities compared to those who were unemployed.

## Discussion

This study assessed DSCA and HRQOL of persons with T2DM in Ho. Most patients with T2DM who reported moderate to severe problems in performing DSCA in this study were significantly high. The study also found that both self-monitoring of blood glucose levels and foot care were poor among the study participants in this study. It is possible that participants were psychologically and emotionally stressed due to the stress associated with being diagnosed with diabetes. Secondly, it may be due to a lack of awareness on the part of the participants to perform diabetes self-care activities. Additionally, T2DM had negative effects on HRQOL. In this study, about 72.0% of the study participants have poor glycaemic control, and they were unable to perform DSCA. This is consistent with previous studies in Ethiopia and China, where patients with poor glycaemic control tend to pay little attention to DSCA [6, 31]. This is possible because cost is a substantial barrier to the use of blood glucose self-monitoring equipment (glucometer) and a healthy diet in Ghana [32]. For instance, the majority of patients with T2DM (73.2%), in this study were unable to afford a glucometer, and therefore their blood glucose levels must be monitored in the hospitals. This also comes with transportation costs, which are also a barrier for accessing these health facilities for monitoring blood glucose levels. However, most of these healthcare facilities are also ill-equipped with glucometers for monitoring blood glucose levels, as reported in an earlier study in Tamale, northern Ghana [33].

Secondly, the proportion of patients who reported moderate to severe problems in performing DSCA in this study was significantly high. This finding is consistent with an earlier study in China [6]. This is possible since many patients may not be aware of the need to initiate DSCA early to avoid complications and psychological stress and its adverse effects on HRQOL.

Also, men were more likely to perform DSCA compared to women. Sex, education and employment significantly influenced the performance of DSCA in this study. This is possible because men tend to engage in more regular physical activities than their women counterparts [2]. Similarly, the majority of patients with diabetes in this study were female. This is similar to previous studies in Ghana that reported a higher prevalence rate of diabetes cases among women compared to men [34–36]. Outside Ghana, the IDF in 2021 also reported a higher prevalence of diabetes cases among women in SSA [1] and in China [37] and the Caribbean [38]. A systematic review by Kazibwe et al. has found that women were more likely to have poor glycaemic control compared to men [36]. This is possible because men tend to engage in moderate-intensity regular physical activity compared to women [2].

Similarly, the study revealed that diabetes education is crucial in performing DSCA. Patients who received diabetes education perform better in DSCA compared to patients who had not received diabetes education. This finding corresponds with an earlier study in northern Ghana [9] where patients with diabetes education were more likely to adhere to DSCA compared to their counterparts with no diabetes education. Also, the results have shown that the educational attainment of patients has a significant difference in the mean score in the performance of DSCA of patients with tertiary education compared to patients with no formal education and junior high school-level education. This finding is in line with previous studies in Ghana [9] and Swedish population-based study [39]. This is possible because formal education enlightens patients on measures to take to prevent diabetes complications compared to patients with no formal education.

Also, the present study has found that the type of employment patients with diabetes engaged in have a significant effect on the performance of DSCA. The findings show that the mean score for patients with public sector employment was significantly higher in performing DSCA compared to self-employed and unemployed patients. Additionally, patients who were employed in the private sector had higher mean scores for self-care activities compared to the unemployed. This is possible because patients who were employed were able to earn income to pay for their healthcare and medications, food, glucometer and machines to engage in regular physical activities.

### Strengths and limitations

Although this study helps explain and expand the literature on the performance of DSCA, the importance of diabetes education on DSCA, and HRQOL, some limitations must be noted. First, the study is limited by the fact that participants were recruited from municipal and teaching hospitals in Ho, so generalisation of the findings should be done with caution.

## Conclusion

The majority of patients with T2DM accessing healthcare at HMH and HTH showed poor diabetes self-care behaviours and HRQOL. Diabetes self-care behaviours could be improved by promoting diabetes education and promoting medication adherence at the health facilities. Also, increasing the frequency of self-monitoring of blood glucose and foot care may improve metabolic and wound control, thereby improving HRQOL.

## Data Availability

The data presented in this study are available and can be obtained from the corresponding author upon reasonable request.

## Abbreviations

DSCA: Diabetes Self–care Activities
HMH: Ho Municipal Hospital
HTH: Ho Teaching Hospital
HRQOL: Health–related Quality of Life
IDF: International Diabetes Federation
T2DM: Type 2 Diabetes Miletus
WHO: World Health Organisation
